# A neonate with left pulmonary artery thrombosis and left lung hypoplasia: a case report

**DOI:** 10.1186/1752-1947-4-284

**Published:** 2010-08-23

**Authors:** Nahed O ElHassan, Christi Sproles, Ritu Sachdeva, Sadaf T Bhutta, Joanne S Szabo

**Affiliations:** 1 Department of Pediatrics, Neonatology, University of Arkansas for Medical Sciences, College of Medicine, Arkansas Children's Hospital, 1 Children's Way, Slot 512-5, Little Rock, AR 72202-3591, USA; 2 Department of Pediatrics, Cardiology, University of Arkansas for Medical Sciences, College of Medicine, Arkansas Children's Hospital, 1 Children's Way, Slot 836, Little Rock, AR 72202, USA; 3 Department of Radiology, Fellowship Director, Pediatric Radiology, University of Arkansas for Medical Sciences, College of Medicine, Arkansas Children's Hospital, 1 Children's Way, Little Rock, AR 72202, USA

## Abstract

**Introduction:**

Spontaneous intrauterine arterial thrombosis and congenital pulmonary hypoplasia are rare conditions and have not been reported to occur together. The literature rather includes two reports of babies with neonatal pulmonary artery occlusion and post-infarction cysts of the lungs.

**Case presentation:**

We report a case of a live Caucasian male newborn with left lung hypoplasia that occurred in association with left pulmonary artery thrombosis. Despite a critical neonatal course, including extracorporeal membrane oxygenation, this infant is alive and well at 18 months of age without any neurodevelopmental sequelae or reactive airway disease.

**Conclusion:**

This association suggests the possibility of an intrauterine vascular event between the fifth and eighth weeks of gestation during early pulmonary artery and lung development.

## Introduction

The prevalence of symptomatic neonatal arterial thrombosis is approximately 1 in 40,000 births, with 90% of cases linked to indwelling intra-arterial catheters [1-4]. Other risk factors are sepsis, polycythemia, maternal diabetes, asphyxia, and inherited thrombophilias [1,3,4]. Very few cases of spontaneous neonatal arterial thrombosis have ever been described [3,4].

Although congenital pulmonary hypoplasia can be idiopathic, it is most commonly associated with conditions that reduce the intrathoracic space [5]. A limited number of reports exist of neonates with congenital pulmonary hypoplasia and no clear evidence of fetal chest compression [5-7]. Two previous reports exist in the literature of neonates with congenital left pulmonary occlusion and postinfarction cysts of the lung [8,9].

We here describe the case of a liveborn male infant with spontaneous intrauterine left pulmonary artery thrombosis and probably associated left lung hypoplasia.

## Case presentation

**Figure 1:**
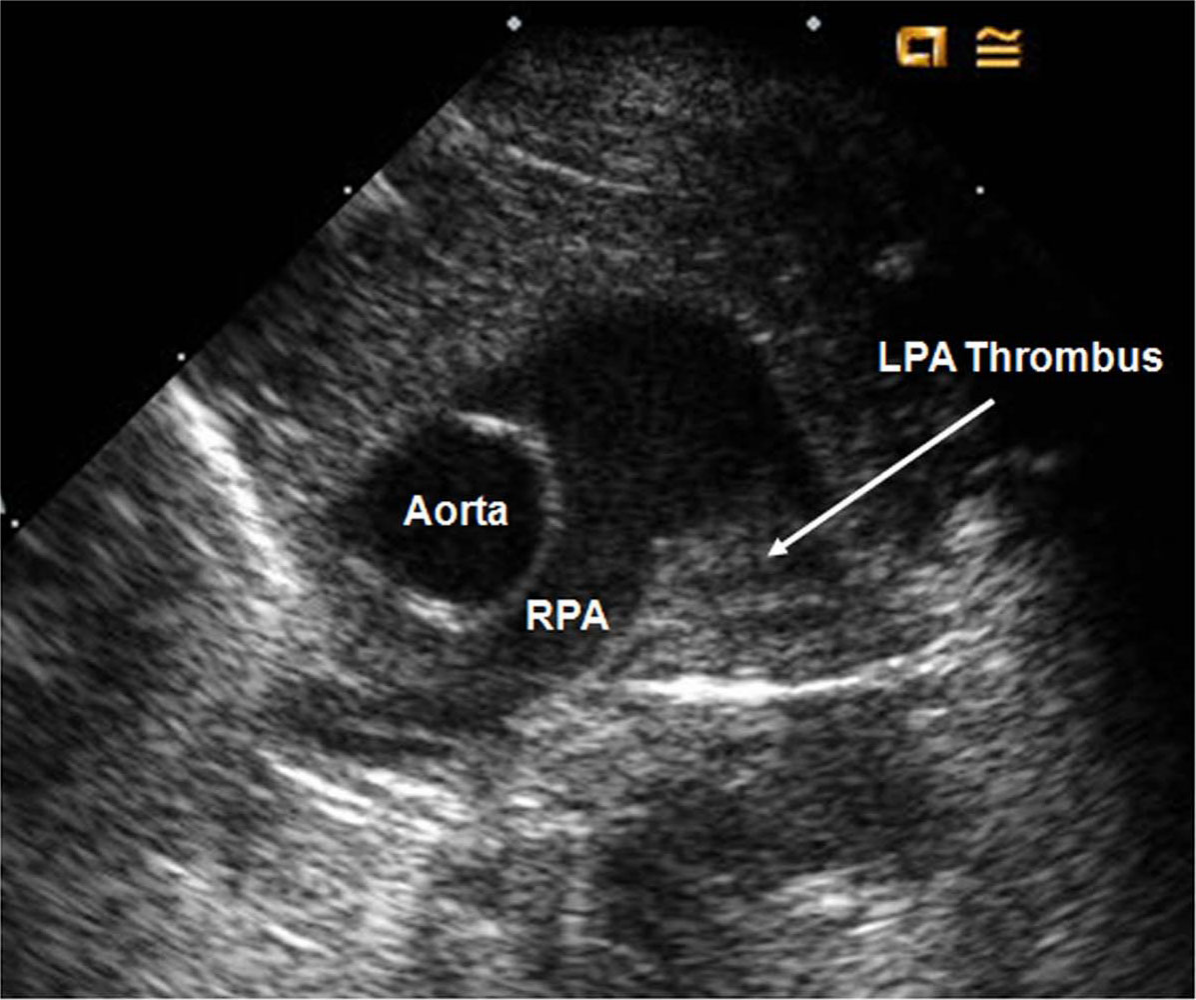
**Cross-sectional view on echocardiogram suggesting the presence of a thrombus in the left pulmonary artery**.

**Figure 2:**
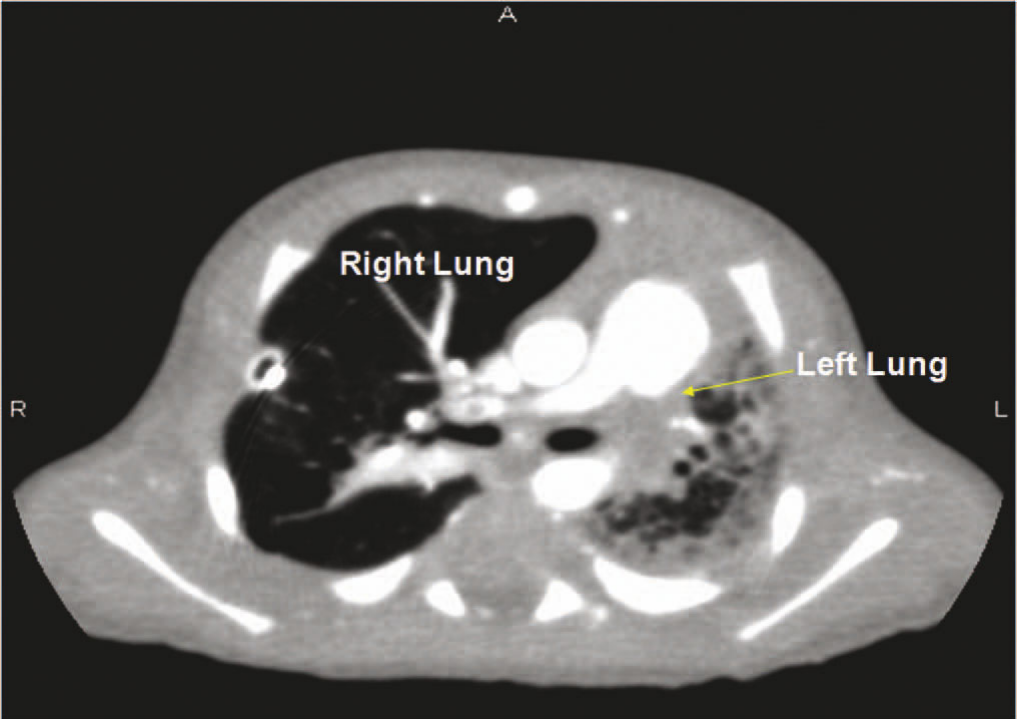
**Cross-sectional view on computed tomography angiography confirming hypoplasia of the left lung**.

**Figure 3:**
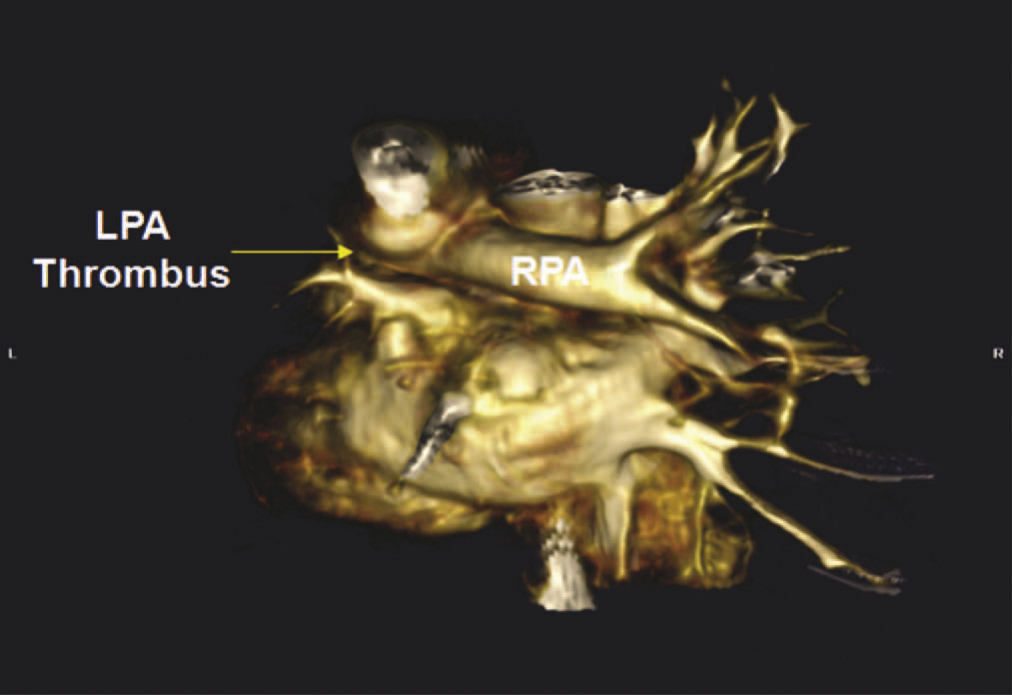
**Three-dimensional view on computed tomography angiography showing a filling defect in the lumen of the left pulmonary artery (LPA) consistent with LPA thrombosis**.

**Figure 4:**
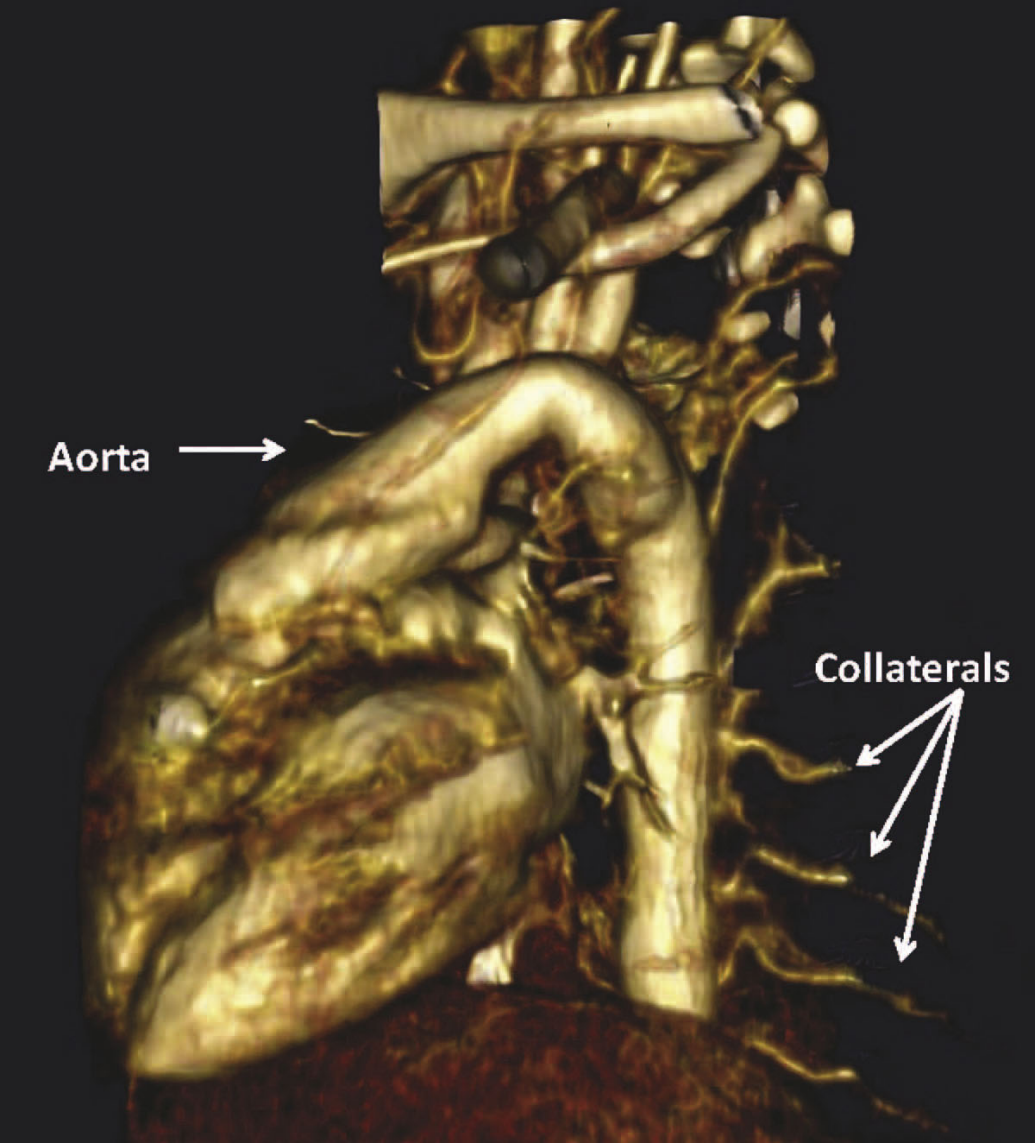
**Three-dimensional view on computed tomography angiography showing collateral vessels originating from the aorta and supplying the left lung**.

A male Caucasian baby was born by spontaneous vaginal delivery at 35 weeks of gestation to a 26-year-old gravida 3, para 1 mother. The mother had well-controlled type 2 diabetes mellitus and two previous miscarriages of unclear etiology. No evidence was found of congenital malformations on prenatal ultrasounds. No family history was known of spontaneous thrombosis. The birth weight was 2353 g and appropriate for gestational age. At 20 minutes of life, he became severely tachypneic. A sepsis evaluation was performed and intravenous antibiotics were begun. Chest radiography revealed a right tension pneumothorax and complete left lung field opacity. The infant was intubated and a chest tube was placed. An echocardiogram at 10 hours of life revealed right ventricular dilatation and hypertrophy with flattening of the ventricular septum, consistent with persistent pulmonary hypertension of the neonate (PPHN). Left pulmonary artery (LPA) blood flow could not be visualized and a thrombus appeared to occlude the LPA (Figure 1). Inhaled nitric oxide was administered. Computed tomography angiography (CTA) at 16 hours of life was performed and showed an intact tracheobronchial tree and a markedly hypoplastic left lung (Figure 2). The main and right pulmonary arteries were normal in caliber, with an occlusion of the LPA by a low-density mass suggestive of a thrombus (Figure 3). In addition, large collateral vessels originated from the distal thoracic aorta and supplied the left lung (Figure 4).

A screening evaluation for a possible inherited thrombophilia disorder was completed in this baby. Blood levels for protein C, protein S, antithrombin activity, concentrations of clottable fibrinogen, plasminogen activity, activities of coagulation factors VIIIC and XII, lipoprotein (a) and homocysteine concentration were within normal limits. DNA-based assays (that is, factor V G1691A mutation and factor II G20210A variant) were also normal [10]. The mother did not complete a thrombophilia screening evaluation. By the third day of life, the baby's oxygenation index was over 40 despite aggressive medical and ventilatory management and he was thus placed on veno-arterial extracorporeal membrane oxygenation (ECMO). Because he had persistent pneumothorax, minimal "rest" ventilator settings were selected. A low positive end-expiratory pressure (0 to 4 cm H_2_O) was maintained for 36 hours until the air leak sealed. The chest tube was removed on day 17 of life. His cardiac and pulmonary function gradually improved on ECMO, allowing decannulation on day 23 of life.

This neonate was heparinized during his ECMO course. Because the management of arterial thrombosis in the neonatal period is controversial, no further anticoagulant therapy was administered after ECMO [11]. He was successfully extubated on day 30 of life. Cranial ultrasounds were normal before and after ECMO.

Because of persistent poor oral motor feeding skills a gastrostomy tube was eventually placed. He was then discharged home on day 128 of life on nasal cannula at 0.5 L/min and 100% oxygen. At discharge, he was taking 50% of his nutrition by bottle, with the rest supplemented via gastrostomy tube. The gastrostomy tube was removed at 9 months of life. The oxygen supplementation was discontinued at 10 months of life. A follow-up echocardiogram at one year of life showed low-velocity flow through the LPA at 0.5 cm/s and LPA hypoplasia. On that date, chest radiography showed interval improvement in left lung aeration and minimal compensatory right lung hyperexpansion.

By 18 months of life, this infant did not require further hospitalizations after his NICU discharge or have clinical evidence of reactive airway disease. In addition, he exhibited age-appropriate neurodevelopment (by Bayley Scales of Infant Development II)

## Discussion

Neonatal arterial thrombosis is, in most cases, iatrogenic from indwelling arterial catheters or lines and is rarely described at birth [2-4]. Some inherited thrombophilia defects, for instance, prothrombotic polymorphisms, Factor V G1691A, Factor II G2021A and the homozygous TT genotype of the methylenetetrahydrofolate reductase (MTHFR) C677T polymorphism, can also increase risk of neonatal arterial thrombosis [10]. Neonatal arterial thrombosis occurs primarily in the aorta and can mimic cyanotic heart disease [1-3]. Two babies were previously described with LPA thrombosis at birth and clinical evidence of PPHN [4,12]. No reported cases were found of infants with congenital LPA thrombosis and left pulmonary hypoplasia.

The pathogenesis of pulmonary hypoplasia is not fully understood, but a normal thoracic cavity volume, adequate respiratory motion and appropriate amniotic fluid volume are all essential for a normal lung growth *in utero *[5,6]. Although congenital pulmonary hypoplasia can sometimes be idiopathic, it typically occurs when any or a combination of these factors is absent or impaired. It has been described in babies with malformations of the chest wall, oligohydramnios and abnormalities of the tracheobronchial tree, although it remains most commonly associated with conditions that reduce intrathoracic space, such as diaphragmatic hernia or pleural effusions [5-7]. Limited reports exist of babies with congenital pulmonary hypoplasia and no clear evidence of fetal lung compression or abnormalities in fetal breathing mechanism or amniotic fluid volume. In those instances, suggested underlying mechanisms are the possibility of a genetic component or a delay in the development of the lung [5,6].

We postulate that a vascular injury could be responsible for the arrest of lung maturation *in utero *and might be the main reason for LPA thrombosis and left pulmonary hypoplasia in this baby. Review of the embryology of the pulmonary vessels indicates that, in this patient, a potential vascular injury and an ensuing pulmonary arterial maturational arrest might have occurred between weeks five and eight of gestation [13]. In the fifth intrauterine week of life, the primitive pulmonary vessels develop from the sixth aortic arch [13]. By week eight of gestation, as the true central pulmonary arteries develop from the aortopulmonary trunk, the primitive pulmonary arteries arising from the aorta involute [13]. It has been previously suggested that the persistence of the aortopulmonary collaterals as the sole source of blood supply to a lobar segment indicates that an intrauterine insult had occurred between weeks five and eight of gestation [13]. We postulate that a vascular injury occurring within this timeline caused an LPA thrombosis and a maturational arrest of the LPA and the left lung.

Another possible explanation is that he had an abnormal left pulmonary vasculature and lung development, and a thrombosis developed later in gestation.

The only identified risk factors for thrombosis in this patient are the maternal history of type 2 diabetes and two previous maternal miscarriages. Of interest, the CTA in this baby identified aortopulmonary collaterals as the primary blood supply to the left lung.

Pulmonary artery thrombosis was first suspected on echocardiogram evaluation. Although cardiac catheterization with contrast angiography may be the gold standard for the diagnosis of arterial thrombosis, CTA is a reliable alternative modality for evaluation of LPA thrombosis [4].

Treatment of neonatal spontaneous arterial thrombosis is controversial. An expert panel on the management of arterial thromboembolic events in neonates recommended that therapy should be individualized based on the extent of thrombosis and the urgency of the clinical situation [11]. Because this patient was stable after ECMO, no further anticoagulant therapy was given.

## Conclusion

In conclusion, this is the first reported case of an intrauterine LPA thrombosis and subsequent pulmonary hypoplasia in a live neonate. This article suggests that a possible vascular injury in the early weeks of gestation is an underlying etiology for such clinical presentation.

## Consent

Written informed consent was obtained from the parents of this patient for publication of this case report and any accompanying images. A copy of the written consent is available for review by the Editor-in-Chief of this journal.

## Competing interests

The authors declare that they have no competing interests.

## Authors' contributions

NEH, CS, RS, STB, and JSS all participated in interpretation, intellectual content, and drafting of the manuscript. All authors have read and approved the manuscript.
